# Indicadores de prescripción racional de medicamentos: factibilidad de aplicación en instituciones de las Américas

**DOI:** 10.26633/RPSP.2021.152

**Published:** 2021-12-22

**Authors:** Cristian Matías Dorati, Perla Mordujovich Buschiazzo, Gustavo H. Marín, Héctor O. Buschiazzo, Robin Rojas-Cortés, María José Alfonso Arvez, José M. Cardozo, Danini Marin, Gilda I. Hernández de Hernández, Noemi Lugo Maldonado, Hugo Marín Piva, José Rego, Sarahan Dussault, Laura Pineda Velandia, Analía Porrás, José Luis Castro

**Affiliations:** 1 Centro Universitario de Farmacología (CUFAR). Universidad Nacional de La Plata La Plata Argentina Centro Universitario de Farmacología (CUFAR). Universidad Nacional de La Plata, La Plata, Argentina.; 2 Organización Panamericana de la Salud Washington DC Estados Unidos de América Organización Panamericana de la Salud, Washington DC, Estados Unidos de América.; 3 Dirección Nacional de Vigilancia Sanitaria Asunción Paraguay Dirección Nacional de Vigilancia Sanitaria, Asunción, Paraguay.; 4 Ministerio de Salud Belmopán Belice Ministerio de Salud, Belmopán, Belice.; 5 Instituto Salvadoreño del Seguro Social San Salvador El Salvador Instituto Salvadoreño del Seguro Social, San Salvador, El Salvador.; 6 Secretaría de Salud Ciudad de México México Secretaría de Salud, Ciudad de México, México.; 7 Caja Costarricense de Seguro Social San José Costa Rica Caja Costarricense de Seguro Social, San José, Costa Rica.; 8 Hospital Docente Dr. Salvador Allende La Habana Cuba Hospital Docente Dr. Salvador Allende, La Habana, Cuba.; 9 Université Laval Quebec Canadá Université Laval, Quebec, Canadá.; 10 Consultora independiente Bogotá Colombia Consultora independiente, Bogotá, Colombia

**Keywords:** Prescripciones de medicamentos, indicadores (estadística), utilización de medicamentos, calidad de la atención de salud, Américas, Drug prescriptions, indicators (statistics), drug utilization, quality of health care, Americas, Prescrições de medicamentos, indicadores (estatística), uso de medicamentos, qualidade da assistência à saúde, Américas

## Abstract

**Objetivo.:**

Evaluar la factibilidad de monitorear la calidad de la utilización de medicamentos en instituciones sanitarias de países de la Región de las Américas mediante indicadores de prescripción racional.

**Métodos.:**

Se realizó un estudio cuantitativo de utilización de medicamentos durante el período 2016-2018. Se desarrollaron y seleccionaron indicadores de prescripción racional de acuerdo a referencias internacionales y a la mejor evidencia disponible para: 1) antiinflamatorios: porcentaje de prescripción de ibuprofeno y/o naproxeno sobre prescripción total de antinflamatorios no esteroideos; 2) antidiabéticos orales: metformina como porcentaje de todos los antidiabéticos prescritos, metformina y/o sulfonilureas como porcentaje de todos los antidiabéticos prescritos; 3) insulinas: insulina cristalina y NPH como porcentaje del total de insulinas prescritas y 4) medicamentos antihipertensivos: porcentaje de inhibidores de la enzima convertidora de la angiotensina (IECA), antagonistas de los receptores de la angiotensina II (ARA-II) y diuréticos tiazídicos sobre el total de antihipertensivos prescritos. Se empleó la dosis diaria definida (DDD) por 1 000 habitantes y día (DHD) como medida del consumo por institución.

**Resultados.:**

La prescripción de metformina con relación a todos los antidiabéticos fue menor al valor del indicador de referencia (27,9%-67,6% vs. 88%), mientras que la prescripción de metformina y/o una sulfonilurea fue comparable con dicho valor (80,9%-97,5% vs. 88%). Los valores de insulina NPH, cristalina y NPH/cristalina con relación a las insulinas prescritas fueron variables frente al valor del indicador de referencia (37,1%-100% vs. 75%). La prescripción de ibuprofeno y naproxeno estuvo por debajo del valor del indicador (20%-50% vs. 80%). El porcentaje de IECA, ARA-II y tiazidas respecto a todos los antihipertensivos osciló entre 65,2%-77,2% vs 65%, acorde al valor del indicador propuesto.

**Conclusiones.:**

Se demostró la factibilidad de aplicar los indicadores de prescripción racional seleccionados y construidos, que proporcionan información útil para analizar la calidad de la prescripción en las instituciones sanitarias de países de la Región y representan una herramienta útil para su monitoreo periódico.

## Abstract (en)

**Objective.:**

Evaluate the feasibility of monitoring the quality of use of medicines in health institutions in countries of the Region of the Americas by means of rational prescription indicators.

**Methodology.:**

A quantitative study of the use of medicines was conducted during the period 2016-2018. Rational prescription indicators were developed and selected in accordance with international reference values and the best available evidence for: 1) anti-inflammatory drugs: prescription of ibuprofen and/or naproxen as a percentage of all prescribed non-steroidal anti-inflammatory drugs; 2) oral antidiabetics: metformin as a percentage of all prescribed antidiabetics, and metformin and/or sulfonylureas as a percentage of all prescribed antidiabetics; 3) insulins: crystalline insulin and NPH as a percentage of total prescribed insulins; and 4) antihypertensive drugs: angiotensin-converting enzyme (ACE) inhibitors, angiotensin II receptor blockers (ARBs-II), and thiazide diuretics as a percentage of all prescribed antihypertensives. The defined daily dose (DDD) per 1 000 inhabitants was used as a measure of consumption per institution.

**Results.:**

Prescription of metformin as a percentage of all antidiabetics was lower than the value of the reference indicator (27.9%-67.6% vs. 88%), while the prescription of metformin and/or a sulfonylurea was comparable with that value (80.9%-97.5% vs. 88%). The values of NPH, crystalline, and NPH/crystalline insulin in relation to all prescribed insulins were variable with respect to the reference indicator (37.1%-100% vs. 75%). Prescription of ibuprofen and naproxen was below the value of the indicator (20%-50% vs. 80%). The percentage of ACE inhibitors, ARBs, and thiazides with respect to all antihypertensives ranged from 65.2%-77.2% to 65%, consistent with the value of the proposed indicator.

**Conclusions.:**

The feasibility of applying the selected and constructed indicators of rational prescription was demonstrated. These indicators provide useful information for analyzing the quality of prescription in health institutions in countries in the Region and are a useful tool for periodically monitoring it.

## RESUMO

**Objetivo.:**

Avaliar a viabilidade de monitorar a qualidade do uso de medicamentos em instituições de saúde em países da Região das Américas por meio de indicadores de prescrição racional.

**Métodos.:**

Foi realizado um estudo quantitativo do uso de medicamentos durante o período 2016-2018. Indicadores de prescrição racional foram desenvolvidos e selecionados de acordo com referências internacionais e as melhores evidências disponíveis para: 1) anti-inflamatórios: prescrições de ibuprofeno e/ou naproxeno como porcentagem da prescrição total de anti-inflamatórios não esteroidais; 2) antidiabéticos orais: prescrições de metformina como porcentagem de todos os antidiabéticos prescritos, metformina e/ou sulfonilureias como porcentagem de todos os antidiabéticos prescritos; 3) insulinas: insulina cristalina e NPH como porcentagem do total de insulinas prescritas; e 4) anti-hipertensivos: porcentagem de inibidores da enzima de conversão da angiotensina (IECA), antagonistas do receptor da angiotensina II (BRA) e diuréticos tiazídicos sobre o total de anti-hipertensivos prescritos. A dose diária definida (DDD) por 1 000 habitantes por dia (DHD) foi usada como medida de consumo por instituição.

**Resultados.:**

A prescrição de metformina em relação a todos os antidiabéticos foi inferior ao valor do indicador de referência (27,9%-67,6% x 88%), enquanto a prescrição de metformina e/ou uma sulfonilureia foi comparável ao valor de referência (80,9%-97,5% x 88%). As porcentagens de prescrição de insulina NPH, cristalina e NPH/cristalina em relação a todas as insulinas prescritas foram variáveis em relação ao valor de referência (37,1%-100% x 75%). A prescrição de ibuprofeno e naproxeno foi inferior ao valor de referência (20%-50% x 80%). A porcentagem de IECA, BRA e tiazídicos em relação a todos os anti-hipertensivos variou entre 65,2% e 77,2%, compatível com o valor do indicador proposto (65%).

**Conclusões.:**

Demonstrou-se a viabilidade de aplicação dos indicadores de prescrição racional aqui selecionados e construídos, os quais fornecem informações úteis para analisar a qualidade da prescrição nas instituições de saúde dos países da Região e representam uma ferramenta útil para seu monitoramento periódico.

El acceso a los medicamentos y otras tecnologías sanitarias y el uso racional de estos constituyen requisitos fundamentales para contribuir al logro de los Objetivos de Desarrollo Sostenible. A pesar de los esfuerzos realizados desde diversos organismos internacionales y países es frecuente observar el uso inapropiado, no efectivo e ineficiente de medicamentos en los servicios de salud (1-2).

Actualmente se continúan desarrollando estudios de utilización de medicamentos en diferentes contextos. Para ello se usan indicadores introductorios que no permiten inferir la calidad de la prescripción de los medicamentos, ya que no exploran todas las dimensiones del uso apropiado de los mismos, ni necesariamente las más importantes (3-5). En este contexto, se hace necesario incorporar herramientas para la evaluación estandarizada de la calidad de las prescripciones en las instituciones o los servicios de salud que proporcionen información sobre la selección prioritaria de los medicamentos con mejor balance beneficio-riesgo-costo, que permitan establecer una relación deseable entre los medicamentos de un grupo terapéutico dado, y brinden datos fundamentales en la toma de decisiones (6-9).

Aunque las experiencias que describen la utilización de relaciones entre medicamentos de un grupo terapéutico como indicadores de racionalidad no son numerosas, estas han demostrado resultados positivos en el uso más eficaz y eficiente de los fármacos con una mejor calidad en la prescripción (6, 10).

Dentro de las principales causas de morbimortalidad en los países de América Latina y el Caribe (ALC) se encuentran enfermedades crónicas como la diabetes mellitus (DM) y las enfermedades cardiovasculares, entre ellas la hipertensión arterial (HTA) (11). La administración y el cumplimiento de los tratamientos en estas enfermedades están lejos de ser óptimos (12). Dentro de los trastornos de salud agudos, los cuadros de dolor e inflamación constituyen un área de indicaciones variables, con una utilización de medicamentos no siempre basada en las mejores evidencias disponibles. Se estima que 30 millones de pacientes utilizan diariamente antiinflamatorios no esteroideos (AINE) en todo el mundo (13).

Por lo expuesto, en este estudio se utilizaron modelos de tratamientos con medicamentos de uso crónico (HTA y DM) y agudo (dolor e inflamación).

El objetivo de este trabajo fue evaluar la factibilidad de monitorear la calidad de la utilización de medicamentos en instituciones sanitarias de países de la Región de las Américas mediante indicadores de prescripción racional.

## MATERIALES Y MÉTODOS

Se realizó un estudio cuantitativo de consumo de medicamentos focalizado en la prescripción mensual y en la compra anual de medicamentos antihipertensivos, antidiabéticos y AINE en servicios públicos de salud con distintas complejidades y niveles territoriales, usando indicadores de calidad de las prescripciones. El estudio se llevó a cabo entre el 1 de enero de 2016 y el 31 de diciembre de 2018.

Se siguió un proceso de selección de indicadores, algunos de ellos construidos en experiencias previas (6-9) y otros de elaboración propia utilizando las evidencias disponibles de los medicamentos con el mejor balance beneficio-riesgo (para el caso de la hipertensión) (14-16). Todos estos indicadores, además, están diseñados para que sean clara y fácilmente comprendidos por los prescriptores y por los profesionales de la salud de las instituciones.

Se tuvieron en cuenta los datos provenientes de siete instituciones de países de la región: Hospital Docente Dr. Salvador Allende, Cuba; Hospital Coatzacoalcos, México; Hospital Regional de Luque, Paraguay; Caja Costarricense del Seguro Social, Costa Rica; Western Regional Hospital, Belice; Instituto Salvadoreño del Seguro Social, El Salvador e Instituto de Obra Médico Asistencial (IOMA), Argentina. Para la presentación de los resultados se asignaron siglas a las instituciones participantes (H1, H2, H3 y H4 representan los hospitales, y S1, S2 y S3 las instituciones de la seguridad social). Los tratamientos para la HTA y la DM se evaluaron en las instituciones H1, H2, S1-S3 y los usados para el dolor y la inflamación en las instituciones H2, H3, H4, S1 y S3.

Para determinar el consumo de medicamentos se incluyeron los datos de los pacientes atendidos en los niveles primario y secundario. Se empleó la dosis diaria definida (DDD) por 1 000 habitantes y día (DHD) como medida del consumo (17), debido a que la DDD es una unidad técnica de medida internacional establecida por la Organización Mundial de la Salud (OMS) (17). Este parámetro permitió estimar el número de personas de cada 1 000 de la población estudiada que estaban recibiendo una DDD por día de los medicamentos seleccionados para este estudio, parámetro común para todos los escenarios

El cálculo de las DHD se realizó mediante la fórmula:


$$\mathrm{DHD}=\frac{\mathrm{mg}\;\mathrm{del}\;\mathrm{fármaco}\;\mathrm{prescritos}\;\mathrm{en}\;\mathrm{un}\;a\tilde{n}o}{\mathrm{DDD}\;\left(\mathrm{mg}\right)\times N^{\circ }\mathrm{de}\;\mathrm{beneficiarios}\;\times 365\;\mathrm{dias}}\times 1\;000$$

Se incluyeron los fármacos comprados y prescritos por cada institución, pero se excluyeron del análisis las formas tópicas (oftálmicas y dérmicas) ya que no se les asigna DDD porque la dosis administrada por día puede variar según la intensidad de la enfermedad. Los datos de compra y prescripción se obtuvieron de los registros de las farmacias pertenecientes a las instituciones participantes. El denominador que se utilizó fue la totalidad de beneficiarios de cada institución participante durante el período de estudio. Este denominador es una de las alternativas propuestas por la OMS para ser utilizadas a nivel hospitalario en caso de no disponibilidad de datos tales como pacientes-días, días de camas ocupadas y egresos (18).

Para la selección de indicadores se realizó una búsqueda en Medline (a través de Pubmed), Cochrane, Tripdatabase y Epistemonikos; revisiones y guías generadas por ministerios de salud de los países de la Región de las Américas y de Europa, la OMS y la Organización Panamericana de la Salud (OPS); y en Google empleando una estrategia y palabras claves definidas. Se priorizó la inclusión de documentos que incluyeran indicadores de calidad de la prescripción de medicamentos. Se consideró, además, cualquier fuente directa o indirecta que mencionara el tema tratado.

A partir de la revisión de información se elaboró una propuesta de indicadores para el tratamiento de cuadros clínicos agudos (inflamación y dolor) (7) y crónicos (diabetes) (6,8,9). Para abordar la prescripción en la HTA se construyó un indicador propio siguiendo el modelo de los indicadores publicados, con base en la referencia de las guías terapéuticas basadas en evidencia y datos del balance beneficio-riesgo de los respectivos medicamentos. El indicador desarrollado incluyó el porcentaje de inhibidores de la enzima convertidora de la angiotensina II (IECA), antagonistas del receptor de la angiotensina II (ARA-II) y/o diuréticos tiazídicos respecto de todos los antihipertensivos prescritos (14-16). El valor propuesto para este indicador es ≥ al 65%.

Para el tratamiento de la inflamación y el dolor, teniendo en cuenta el uso de indicadores en experiencias previas (7) corroborado por evidencias sobre beneficio-riesgo (13, 19), se decidió emplear como indicador la relación de uso de ibuprofeno y/o naproxeno como porcentaje de todos los AINE prescritos. El valor del indicador es ≥ 80%. (7)

Para la diabetes se consideró el porcentaje de prescripción de metformina respecto de todos los hipoglucemiantes prescritos (Valor referido en la bibliografía es ≥ 88% (8)), el de metformina y/o sulfonilureas como porcentaje de todos los hipoglucemiantes prescritos (Valor referido en la bibliografía es ≥88% (9)), y el de insulina NPH y/o cristalina como porcentaje de todas las insulinas prescritas (Valor referido en la bibliografía es ≥ 75% (6)). Estos indicadores se seleccionaron con base en su uso en experiencias previas (8, 9) y evidencias de beneficio-riesgo de los respectivos medicamentos, inclusive una disminución de la mortalidad con el empleo de metformina (20-27). Con respecto a las insulinas, no existen estudios de superioridad que demuestren un efecto beneficioso en eficacia y seguridad del uso de análogos frente a la insulina NPH. Si bien existen reportes que muestran menores tasas de hipoglucemia con análogos de la insulina, no hay información concluyente de diferencias en el número de muertes por hipoglucemias severas (6, 28-32). Además, los análogos de la insulina no resultaron costo-efectivos en evaluaciones independientes, carentes de sesgo (29-33). Por lo anterior, se seleccionó la utilización de insulina cristalina y/o NPH como porcentaje del total de insulinas prescriptas como indicador de prescripción racional (6).

## RESULTADOS

Los patrones de utilización de antihipertensivos en las instituciones de salud evaluadas (H1, H2 y S1-S3), por subgrupo terapéutico y principio activo se muestran en el cuadro 1. En los hospitales, los IECA son los antihipertensivos más prescritos. En el grupo de instituciones de la seguridad social la prescripción de IECA o ARA-II es mayor al 30%, salvo en una institución (S1) donde los ARA-II se prescriben en un 17%, porcentaje comparable a la prescripción de y los bloqueantes de los canales de calcio en dicha institución.

**CUADRO 1.** Consumo de antihipertensivos por institución (DHD)

Medicamentos	Instituciones				
	H1	H2	S1	S2	S3
Enalapril	30,85	141,25	100,14	91,18	36,09
Lisinopril	0	0	0	0	0,31
Ramipril	0	0	0	0	0,38
Captopril	4,71	0	0,0001	1,32	0
**Subtotal IECA**	**35,56**	**141,25**	**100,14**	**92,5**	**36,78**
Candesartan	0	0	0	0	2,38
Irbesartan	0	0	49,06	65,38	0,59
Losartan	6	8,22	0	0	17,51
Telmisartan	0	0	0	0	5,05
Valsartan	0	0	0,0007	0	10,89
**Subtotal ARA-II**	**6**	**8,22**	**49,06**	**65,38**	**36,42**
Amlodipina	6,8	24,05	48,12	25,76	14,19
Nifedipina	1,87	0,18	0	0	0,65
Nifedipina retard	0	0	0	0	0,2
Diltiazem	0,14	0	0,0015	0	1,05
Verapamilo	0,34	0	1,15	3,61	0,13
**Subtotal bloqueantes cálcicos**	**9,15**	**24,23**	**49,27**	**29,37**	**16,22**
α-metildopa	0,75	2,67	1,76	0,24	0
Clonidina	0	0	0	0,14	0
**Subtotal acción central**	**0,75**	**2,67**	**1,76**	**0,38**	**0**
Atenolol	2,88	7,05	23,22	7,7	6,86
Bisoprolol	0	0	0	0	5,04
Metoprolol	0	0	0	0	0,15
Carvedilol	0	4,84	0,98	2,27	2,5
Nebivolol	0	0	0	9,12	0
Labetalol	0,00028	0,13	0	0	0
Propranolol	0,21	0,26	0,96	1,06	0,25
**Subtotal betabloqueantes**	**3,09**	**12,28**	**25,16**	**20,15**	**14,8**
HCTZ	6,29	6,82	39,93	15,49	7,8
Clortalidona	0	0	0	0	0,91
**Subtotal tiazidas**	**6,29**	**6,82**	**39,93**	**15,49**	**8,71**
Espironolactona	0,48	0	3,33	0	0
Furosemida	4,26	5,86	16,94	6,88	2,33
Nitroprusiato de sodio	0	0	0,001	0	0
Hidralazina	0,1	0	0,49	0,41	0,002
Enalapril+HCTZ	0	1,14	0	0	0,0005
HCTZ+Amilorida	0	0	0	0	3,01
Losartan+HCTZ	2,19	0	0	0	0
Amlodipina+HCTZ+valsartán	0	0	0	17,78	0
Amlodipina+valsartán	0	0	0	17,51	0
**Subtotal otros**	**7,03**	**7,00**	**20,76**	**42,58**	**5,34**
**TOTAL DHD**	**67,87**	**202,47**	**286,08**	**265,85**	**118,27**

n=número de pacientes totales atendidos en el mes de registro; N= número de pacientes bajo cobertura durante el período en que se registraron las compras; H1: n=19 659 (junio 2018); H2: n=30 514 (noviembre 2017); S1: N=4 500 000 (anual, 2017); S2: N=1 600 000 (junio 2018); S3: N=2 034 180 (anual, 2016); DHD: dosis diaria definida (DDD) por 1 000 habitantes y día; IECA: inhibidores de la enzima convertidora de la angiotensina; ARA-II: antagonistas de los receptores AT 1 de angiotensina II; HCTZ: hidroclorotiazida.

**FIGURA 1.**
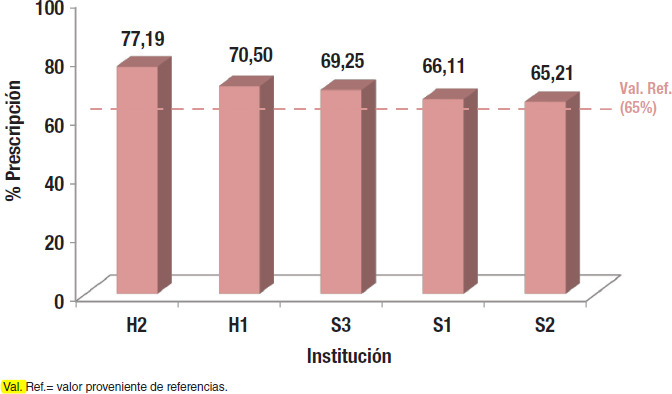
Porcentaje de IECA, ARA-II y/o tiazidas en relación con el consumo total de antihipertensivos en las instituciones analizadas

Val. Ref.= valor proveniente de referencias.

En relación a la evaluación de la calidad de prescripción en las instituciones participantes, el porcentaje de prescripción de medicamentos del grupo IECA, ARA-II y/o tiazidas respecto de todos los antihipertensivos prescriptos osciló entre 65,21 % y 77,19 % (Figura 1), un valor comparable al propuesto para el indicador (65%).

**FIGURA 2.**
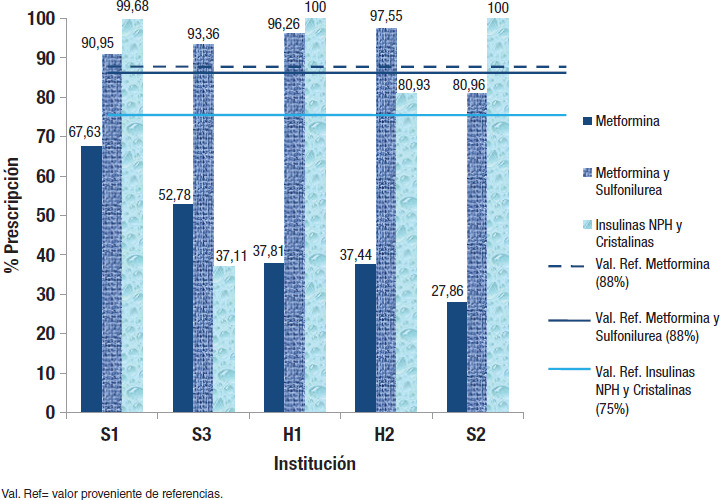
Porcentaje de prescripción de metformina, metformina y sulfonilureas e insulinas NPH y cristalina en relación con el consumo total de antidiabeticos en las instituciones analizadas

Los patrones de utilización de antidiabéticos por subgrupo terapéutico y principio activo en las instituciones evaluadas (H1, H2 y S1-S3) se muestran en el cuadro 2. En las instituciones hospitalarias se prescribe alrededor de un 60% de sulfonilureas de larga duración de acción, casi un 40% de metformina y <4 % de insulinas. En las instituciones de la seguridad social se prescribe mayoritariamente metformina (entre 52,78% y 67,63%), 23% a 40% de sulfonilureas y 6% a 19% de insulinas. En la evaluación de la calidad de la prescripción para este grupo de medicamentos, el porcentaje de prescripción de metformina de las instituciones con relación a la prescripción de todos los antidiabéticos varió entre el 27,86 % y el 67,63%, valores por debajo del indicador de referencia (88%). El porcentaje de prescripción de metformina y/o una sulfonilurea varió entre 80,96 % y 97,55%, valores que se corresponden con el indicador de referencia (88%) (Figura 2). Con respecto a las insulinas, el porcentaje de insulina NPH, insulina cristalina y NPH/cristalina en relación con todas las insulinas prescritas se encuentra entre 37,11% y 100 %, un rango amplio en relación con el indicador de referencia (75%) (Figura 2).

**FIGURA 3.**
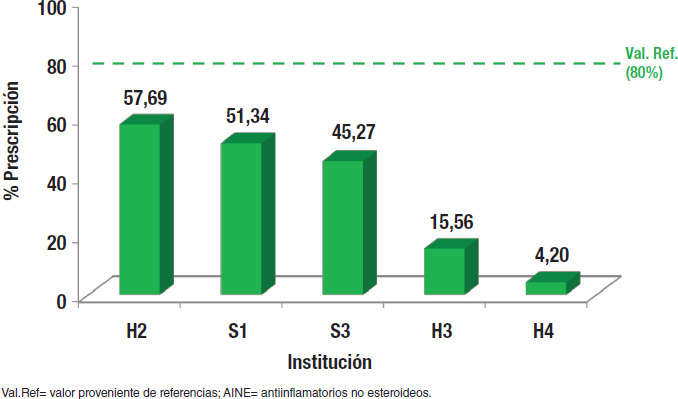
Porcentaje de prescripción de ibuprofeno y/o naproxeno en relación con el consumo total de AINE en las instituciones analizadas

En el cuadro 3 se muestran los patrones de utilización de AINE por principio activo en las instituciones evaluadas (H2-H4 y S1, S3). Solamente en uno de los hospitales participantes se prescribe mayoritariamente ibuprofeno (57,69%). En los restantes se utilizan principalmente medicamentos del grupo coxib o diclofenaco. En las instituciones de la seguridad social, la prescripción mayoritaria de AINE correspondió al ibuprofeno (>40%). Al hacer la evaluación de la calidad de la prescripción para estos fármacos observamos que en las instituciones participantes se obtuvieron valores de prescripción de ibuprofeno y naproxeno por debajo del valor del indicador tomado como referencia (80%). En las instituciones H2, S1 y S3 este valor fue de alrededor del 50%, y en H3 y H4 se ubicó por debajo del 20% (figura 3).

**CUADRO 2.** Consumo de hipoglucemiantes orales e insulina por institución (DHD)

Medicamentos	Instituciones				
	H1	H2	S1	S2	S3
Metformina	7,58	16,52	25,64	27,83 (7,73 en CDF con glimepirida)	12,76
**Total metformina**	**7,58**	**16,52**	**25,64**	**27,83**	**12,76**
Glimepirida	0	26,52	0,0001	53,12 (25,91 en CDF con metformina)	3,75
Glibenclamida	11,72	0	8,84	0	1,31
Glipizida	0	0	0	0	0,06
Gliclazida	0	0	0	0	4,69
**Total sulfonilureas**	**11,72**	**26,52**	**8,84**	**53,12**	**9,81**
Pioglitazona	0	0	0	0	0,021
Rosiglitazona	0	0	0	0	0,002
**Total glitazonas**	**0**	**0**	**0**	**0**	**0,02**
Linagliptina	0	0	0	0	0,03
Saxagliptina	0	0	0	0	0,026
Sitagliptina	0	0,15	0	0	0,00014
**Total inhibidores DPP4**	**0**	**0,15**	**0**	**0**	**0,06**
Exenatida	0	0	0	0	0,009
Liraglutida	0	0,03	0	0	0,001
**Total agonistas GLP1**	**0**	**0,03**	**0**	**0**	**0,01**
Repaglinida	0	0	0	0	0,005
**Total DHD orales**	**19,30**	**43,22**	**34,48**	**80,95**	**22,66**
Insulina cristalina humana	0,025	0,13	0,78	2,56	0,058
**Total insulina cristalina humana**	**0,025**	**0,13**	**0,78**	**2,56**	**0,058**
Insulina lispro	**0**	0,028	0,0061	**0**	0,12
Insulina glulisina	**0**	**0**	0,00008	**0**	0,044
Insulina aspártica (insulina humana aspartato)	**0**	**0**	**0**	**0**	0,39
**Total análogos acción corta**	**0**	**0,03**	**0,01**	**0**	**0,55**
Insulina NPH	0,32	0,51	2,64	16,48	0,5
**Total insulina NPH**	**0,32**	**0,51**	**2,64**	**16,48**	**0,5**
Insulina glargina	**0**	0,11	0,0045	**0**	0,26
Insulina detemir	**0**	0,009	**0**	**0**	0,093
Insulina degludec	**0**	0,025	**0**	**0**	**0**
**Total análogos acción prolongada**	**0**	**0,14**	**0,0045**	**0**	**0,35**
Insulina lispro bifásica (lispro+lispro protamina)	**0**	**0**	0,00015	**0**	0,019
Insulina aspártica bifásica (Insulina HM bifásica aspartato)	**0**	**0**	**0**	**0**	0,025
**Total bifásicas (análogos)**	**0**	**0**	**0,00015**	**0**	**0,04**
Insulina NPH/cristalina	0,4	0,09	**0**	**0**	0,0032
**Total DHD insulinas**	**0,75**	**0,90**	**3,43**	**19,04**	**1,51**
**Total DHD antidiabéticos**	**20,05**	**44,12**	**37,91**	**99,99**	**24,17**

n= número de pacientes totales atendidos en el mes de registro, N= número de pacientes bajo cobertura durante el período en que se registraron las compras, H1: n=19 659 (junio 2018); H2: n=30 514 (noviembre 2017); S1: N=4 500 000 (anual, 2017); S2: N=1 600 000 (junio 2018); S3: N=2 034 180 (anual, 2016); DPP4: dipeptidil peptidasa-4; GLP1: péptido similar al glucagón-1; DHD: DDD/1 000 habitantes/día; CDF: combinación en dosis fijas.

## DISCUSIÓN

En una primera evaluación de los resultados se observó que en la mayoría de las instituciones participantes se registraron valores de consumo de los antihipertensivos, metformina, sulfonilureas e insulina cristalina y NPH que se adecuaban a los indicadores propuestos, con mayores oportunidades de mejora en el uso de naproxeno e ibuprofeno dentro del grupo de los AINE.

Para el tratamiento de la HTA, en todas las instituciones el perfil de prescripción observado coincidió con el de los medicamentos de mejor relación beneficio-riesgo. En todos los casos se utilizan fármacos pertenecientes a los grupos de IECA, ARA-II o diuréticos tiazídicos en una proporción que se correlaciona con el indicador propuesto (> 65%), lo que concuerda con los datos de utilización reportados en otros entornos en los cuales este porcentaje fue de 68,2% y 66,3% (34). Este resultado, si bien no idéntico, se aproxima al valor más alto descrito en España (73,8%) (35).

Val. Ref= valor proveniente de referencias.

**CUADRO 3.** Consumo de antiinflamatorios no esteroideos por institución (DHD)

Medicamentos AINE	Instituciones				
	H2	S1	S3	H3	H4
Celecoxib	0	0	0,0002	0	32,64
Diclofenaco	14,59	1,04	3,01	61,14	0
Etoricoxib	0	0	2,56	0	6,61
Ibuprofeno	20,45	12,8	5,52	8,69	3,53
Indometacina	0	2,72	0,24	1,45	9,95
Ketorolaco	0,41	0	1,07	0	12,93
Meloxicam	0	0	0,93	0	18,41
Naproxeno	0	0	1,05	2,99	0
Piroxicam	0	0	0,002	0,78	0
Sulindaco	0	6,18	0	0	0
Tenoxicam	0	2,19	0	0	0
Dexketoprofeno	0	0	0,13	0	0
AAS	0	0	0,0017	0	0
**Total (DHD)**	**35,45**	**24,93**	**14,51**	**75,05**	**84,07**

H3: n=859 (junio 2016); H4: n= 5 504 (julio 2016); H2: n=31 467 (julio 2016); S1: N=4 500 000 (anual, 2017); S3: N=2 034 180 (anual, 2016); AAS: ácido acetilsalicílico; DHD: DDD/1 000 habitantes/día; AINE= antiinflamatorios no esteroideos.

Considerando que los diuréticos tiazídicos continúan siendo medicamentos de elección por su efectividad para prevenir complicaciones cardiovasculares, su buen perfil de seguridad a las dosis bajas recomendadas y el bajo costo, es llamativa su baja utilización (3%-14%), por debajo de los agentes bloqueantes de los canales de calcio (11%-26%). Este resultado se encuentra en consonancia con la tendencia de uso de estos medicamentos observada en un estudio llevado a cabo entre 2005 y 2016 (36) y difiere de las informadas en un estudio de 2014 (37) según el que, entre los pacientes hipertensos, el uso de tiazidas alcanzó el 24%, mientras que el uso de bloqueantes de los canales de calcio fue cercano al 20%.

Val. Ref= valor proveniente de referencias; AINE= antiinflamatorios no esteroideos.

Es importante remarcar que en los hospitales analizados (H1, H2), la prescripción de furosemida fue cercana a la de diuréticos tiazídicos, lo cual sugeriría una oportunidad de mejora en la reducción del consumo de furosemida, una vez que se revise la proporción de pacientes hipertensos con insuficiencia cardíaca que lo podrían requerir.

En el caso de los antidiabéticos, la tasa de prescripción de metformina encontrada en el estudio fue baja (entre 27,86 % y 67,63%), a pesar de ser el medicamento de primera línea para la mayoría de los pacientes diabéticos (21-23). Entre las sulfonilureas, la gliclazida o la glipizida constituyen las mejores alternativas ya que no producen metabolitos activos y el sistema enzimático del citocromo no interviene en su metabolización (38). Lo anterior se contrasta con que en tres de las cinco instituciones evaluadas (H1, H2 y S2), la glimepirida o la glibenclamida se prescribieron más que la metformina (57% vs 34% en promedio, respectivamente), lo que sugiere un patrón invertido de prescripción racional, con mayor riesgo de hipoglucemia e interacciones medicamentosas en el tratamiento de los pacientes diabéticos polimedicados. Solo dos instituciones (H2 y S3) reportaron una prescripción muy baja (0,4%) de medicamentos que no cuentan con evidencia de alta calidad que demuestre su efectividad y potencial prevención de complicaciones. En los casos en que es necesario incorporar al tratamiento insulina, las formas NPH y cristalina constituyen las de primera elección ya que el uso de análogos de la insulina no ha demostrado ser costo-efectivo (29). En nuestro estudio se observó que en cuatro instituciones (H1, H2, S1, S2) se prefiere el uso de las primeras por encima del 75%, mientras que en una de ellas (S3), 6 de cada 10 pacientes reciben análogos de la insulina de acción corta y prolongada, lo que constituye una estrategia no costo-efectiva (6, 29).

En relación con el uso de AINE no existe evidencia de que algún medicamento del grupo posea una eficacia superior al resto; no obstante, existe una amplia diferencia entre sus perfiles de seguridad, que debe considerarse en su selección junto con los factores de riesgo que presente el paciente (13,19,). Por ejemplo, el meloxicam, el ketorolaco y la indometacina presentarían un mayor riesgo de lesión gastrointestinal y renal con respecto al ibuprofeno (39,40,). En las instituciones participantes se observó entre el 43% y el 96% de la prescripción de medicamentos del grupo AINE con una relación beneficio-riesgo desfavorable, y por lo tanto estaría lejos de cumplirse el valor del indicador seleccionado (7).

En la institución H4, cerca de la mitad de la prescripción de AINE correspondió al etoricoxib y el celecoxib, mientras que la prescripción de ibuprofeno fue muy baja y la de naproxeno nula. En la institución H3, el diclofenaco fue el medicamento más prescrito, y medicamentos con mejor perfil de seguridad, como el ibuprofeno y el naproxeno, se prescribieron en segundo y tercer lugar. El uso de ibuprofeno fue mayoritario solo en tres de las cinco instituciones analizadas (H2, S1 y S3). Sin embargo, en ningún caso sobrepasó el 50% de los AINE prescritos, y otras opciones con un perfil de seguridad desfavorable ocuparon un lugar importante en la prescripción.

En este estudio se analizaron indicadores que establecen relaciones de uso o consumo de medicamentos de un mismo grupo terapéutico en servicios de salud, como forma indirecta de monitorear la prescripción (desenlace subrogado) (41). Esto permitió evaluar aspectos más concretos de la prescripción que los obtenidos utilizando los indicadores de uso de medicamentos propuestos por la OMS, que siguen siendo utilizados actualmente (3-5). El uso de este tipo de indicadores de prescripción ha permitido a países como Escocia (42) y Gales (43) contar con una herramienta para optimizar el uso de los medicamentos.

Los valores obtenidos en este estudio para los indicadores de calidad de la prescripciones propuestos permiten identificar patrones de consumo de medicamentos en enfermedades prioritarias en algunas instituciones de América Latina y el Caribe y valorar su utilización frente a recomendaciones farmacoterapéuticas basadas en la evidencia. Estos indicadores, además, pueden emplearse para monitorear periódicamente el uso de los medicamentos en las instituciones sanitarias y deberán actualizarse en función de los medicamentos disponibles en el mercado y las evidencias actualizadas sobre beneficio-riesgo y costo. La implementación de este tipo de indicadores a nivel institucional requerirá un acuerdo, no solo de las autoridades de la institución o del servicio, sino de todo el equipo de salud.

Este estudio tiene algunas limitaciones. Primero, no fue posible contrastar los patrones de uso de medicamentos según las indicaciones específicas para la población de estudio debido a que las instituciones participantes no registran el diagnóstico para el que son utilizados. Segundo, los valores tomados como referencia para los indicadores provienen de datos de la literatura y de otros contextos puesto que no existían marcos o estándares de comparación en las instituciones. En tercer lugar, en algunos casos se tomaron datos indirectos de prescripción a partir de compras de medicamentos. Finalmente, el uso de períodos de evaluación diferentes limita la comparación entre las instituciones.

En conclusión, este estudio demuestra la factibilidad de utilizar nuevos indicadores de prescripción racional, aplicables en forma sistemática al monitoreo del consumo de medicamentos en instituciones sanitarias, que brindan información acerca de aspectos de la prescripción fundamentales para la toma de decisiones y el establecimiento de estrategias que permitan optimizar el uso de medicamentos en los servicios de salud.

## Declaración.

Las opiniones expresadas en este manuscrito son responsabilidad de los autor y no reflejan necesariamente los criterios ni la política de la *RPSP/PAJPH* o de la OPS.
